# Creating a Window into the Developing Brain: Observing Axon Growth in Live Mice

**DOI:** 10.1371/journal.pbio.0030301

**Published:** 2005-07-26

Soon after a human baby emerges from the womb, its brain churns out new cells at an unbelievable rate of 250,000 neurons per minute. Each neuron typically acquires multiple dendrites to pick up electrical signals from other cells, and just one axon to transmit those signals to other cells. An axon can grow several feet in the service of relaying signals to its target tissue, but how it manages this growth has been an open question. Now Carlos Portera-Cailliau and colleagues use the real-time fidelity offered by two-photon microscopy to peer inside the brain of a living neonatal mouse and observe axons growing and reorganizing. By creating a window into the developing mouse brain, the authors could watch and record time-lapse images of living axons as they migrated through the neural landscape. [Fig pbio-0030301-g001]

**Figure pbio-0030301-g001:**
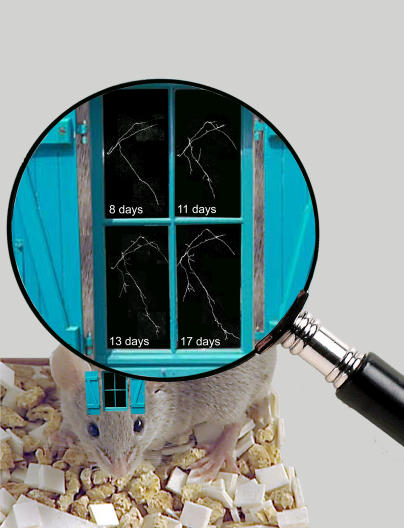
Implanting “windows” into the skulls of newborn mice allowed researchers to observe axon development

Researchers have long been aware that a complex process of axon growth and retraction occurs during the first few weeks of life, but until now have not been able to observe it for themselves in real time, at least not in mammals. Axon growth and guidance have been studied extensively in cultured neurons, and, along with time-lapse imaging of the brains of live frogs and fish, have provided insights into brain development in lower vertebrates. But because studies of mammalian axon growth have relied on measurements taken from fixed brain tissue samples, very little is known about the details of the growth and refinement of axonal projections in mammals.

Portera-Cailliau et al. solved this problem by using two-photon microscopy, a state-of-the-art imaging technique that allowed them to watch the same developing axons in the cerebral cortex of live mice during the first two weeks of life. The group used a line of transgenic mice that expressed green fluorescent protein in small subsets of neurons. The procedure involved removing a portion of the skull and replacing it with a glass coverslip, thus creating a window for viewing live brain tissue at different developmental stages. Although traditional confocal microscopy can also be used to view fluorescently labeled cells, two-photon microscopy can image deeper into tissue without damaging the brain cells, which allows imaging repeatedly over long periods of time.

Time-lapse images were generated from the mice over a period of several minutes to as long as three weeks, allowing the researchers to observe changes in neuronal structure throughout the critical period of cortical development. Portera-Cailliau et al. observed changes in two very different types of neurons: thalamocortical (TC) neurons and Cajal-Retzius (CR) neurons. Both of these neurons send axons to the outermost layer of the cortex, but CR neurons project only locally and TC neurons project over long distances. The structure and dynamics of axons from these neurons, it turns out, are also very different, indicating that axonal development is not homogeneous across cell types. For example, TC axons grew quickly in long, straight paths and added new branches frequently. By contrast, CR axons grew much more slowly, along tortuous paths. Additionally, TC axons exhibited both short branch retraction (tens of microns) and elimination of larger branches (hundreds of microns or more), while CR axons only used branch tip retraction for pruning. Thus, the structure and dynamics of axon elaboration are dependent on neuronal cell type, even when they grow side by side in the same environment. This suggests that different neurons may exhibit different axon elaboration programs and/or interpret differently cues from their surroundings.

This study provides insight into the process of refinement and optimization of neuronal circuitry that occurs in mammals in the early stages of life, and begins to solve the mystery about how axons develop in the cortex. By observing axon growth in real time, scientists have taken the first step in understanding the cues that control each twist and turn of every axon in the cortex. Such efforts may one day suggest ways to prevent or treat the many types of cognitive disabilities that arise from abnormalities in brain development.

